# Editorial: Toolkits for Prediction and Early Detection of Acute Exacerbations of Chronic Obstructive Pulmonary Disease

**DOI:** 10.3389/fmed.2022.899450

**Published:** 2022-04-27

**Authors:** Yahong Chen, Jing Zhang, Jeffrey L. Curtis

**Affiliations:** ^1^Department of Pulmonary and Critical Care Medicine, Peking University Third Hospital, Beijing, China; ^2^Department of Pulmonary and Critical Care Medicine, Shanghai Medical College, Zhongshan Hospital, Fudan University, Shanghai, China; ^3^Pulmonary and Critical Care Medicine Division, Department of Internal Medicine, University of Michigan, Ann Arbor, MI, United States; ^4^Medical Service, VA Ann Arbor Healthcare System, Ann Arbor, MI, United States

**Keywords:** chronic obstructive pulmonary disease, biomarkers, blood eosinophil, COVID-19, *Aspergillus*, microbiome

Chronic obstructive pulmonary disease (COPD) is an inflammatory chronic airway disease with high morbidity and mortality, plus serious social and economic burden (1, 2). Acute exacerbations (AECOPD) are periods of worsened symptoms, outside a given patient's day-to-day fluctuations, of dyspnea and cough with increased sputum production, often accompanied by fatigue, sleep disturbance, and sputum purulence. AECOPD are a critical prognostic indicator of future risk, as they can reduce quality of life, increase hospitalization rate, accelerate disease progression, and even result in death (3).

Due to this reliance on subjectively greater symptoms, defining AECOPD remains controversial (4). In the real world, COPD patients are often unable to express fully their daily symptom fluctuations and the range of recent changes. Additionally, it can be difficult for respiratory physicians to tell with certainty if the patient's description is really more than the “bad days” that virtually all COPD patients describe (5). Hence, definition of all but the most severe AECOPD lacks clinical operability, which may delay diagnosis and proper treatment, risking more serious clinical outcomes. Many clinical therapeutic trials accordingly use a “healthcare resource utilization” definition as their outcome; in other words, an exacerbation is defined by an encounter with the healthcare system that led to a change in medications and site-of-care. However, although objective, this definition depends heavily on access to care, and may also miss alternative serious causes of respiratory symptoms.

Because early identification of AECOPD must be central to COPD care, more effective tools are needed. Two alternative approaches are quantification of patient reported outcomes (PRO) (6) and measurement of biomarkers (7). To date, questionnaires in use include EXACT, CAT, BCSS, and daily diary cards. Studies on them are on-going, with the aim of a suitable tool for point-of-care use, especially in primary care settings, to help physicians to detect AECOPD early. However, questionnaire cutoffs are inconsistent between studies, and only some of the events that they identify are clinically significant. Progress in telehealth could help to detect symptom changes remotely and trigger an alert to intervene. Defining reliable predictive biomarkers would be a clinically significant achievement in early diagnosis and management of AECOPD, but we still have a long way to go there. Biomarkers in a broad sense include physical parameters such as breath rate and PEF besides blood and/or sputum indicators.

In the special Research Topic, “*Toolkits for Prediction and Early Detection of Acute Exacerbations of Chronic Obstructive Pulmonary Disease”*, three articles are related to predictive models for AECOPD risk, and another three highlight new etiological entities causing AECOPD. This Research Topic aims to support the prediction of AECOPD and to provide guidance on individualized therapy in AECOPD.

## Predictive Models for AECOPD Risk

Blood eosinophils have been used to identify populations at high-risk of AECOPD and to guide corticosteroid therapy. However, the value of blood eosinophils in hospitalized AECOPD remains controversial, especially lacking large sample data from China, the country with the world's greatest burden of COPD. The study by Cui et al. aimed to evaluate the accuracy of eosinophils to predict clinical outcomes in AECOPD. The analysis uses data from the ACURE study, an ongoing nationwide multicenter, observational, pragmatic study in patients admitted for AECOPD. Based on a threshold of blood eosinophils at 2% of the total leukocyte count, 42.7% had an eosinophilic AECOPD. After propensity score matching, elevated eosinophils were only associated with better short-term outcomes in patients with a smoking history. These results suggest that eosinophil levels alone cannot be confidently used as a predictor to estimate prognosis.

AECOPD affect patients' health and can lead to death. The study by Dong et al. developed a prediction model for in-hospital mortality in patients with AECOPD. Four variables were included into the final model: age, respiratory failure, pneumothorax, and length-of-stay. Importantly, this simple prediction model for in-hospital mortality during AECOPD was externally validated. Complications emerged as strong predictors, underscoring an important role of disease management in improving patients' prognoses during exacerbation episodes. Based on available data in clinical setting, the model could serve as an easily used instrument for clinical decision-making.

The final study in this group, from Liu et al., determined the utility in clinical practice of hemogram indexes indicating systemic inflammation to predict hospitalization and mortality in AECOPD. They examined associations of AECOPD with platelet–lymphocyte ratio (PLR), platelet×neutrophil/lymphocyte ratio [systemic immune-inflammation index (SII)], and monocyte×neutrophil/lymphocyte ratio [systemic inflammation response index (SIRI)]. Combining inflammatory hemogram index PLR with other indexes was a promisingly simple and effective marker to predict exacerbation in patients with stable COPD.

## New Etiological Entities Causing AECOPD

Abnormalities in lower respiratory tract microbiome increase the risk of AECOPD. It is well acknowledged that bacterial overload contributes to the chronic airway inflammation. More recently, an increased interest has been aroused in the impact of airway *Aspergillus* on exacerbation vulnerability. *Aspergillus* species can be identified in sputum samples during both moderate and severe AECOPD, although their clinical relevance remains unclear. In a retrospective cohort of hospitalized AECOPD from eight centers, Wu et al. found that A*spergillus* colonization may predict poor prognosis of AECOPD, while also leading to an increased short-term risk of recurrent AECOPD.

The COVID-19 pandemic has made routine management and diagnosis of COPD more challenging due to reduced face-to face consultations, difficulties in performing spirometry, and limitation in traditional pulmonary rehabilitation and home care programs. Patients with COPD are at an increased risk of hospitalization for COVID-19 and may be at increased risk of developing severe disease and death. Polverino and Kheradmand reviewed the epidemiological, immunological, and clinical aspects. SARS-CoV-2 shares common pathobiological and clinical features with other viral agents responsible for increased morbidity, thus representing a novel cause of AECOPD with the potential for a more long-term adverse impact.

Systemic corticosteroids (SCS) can improve lung function (FEV_1_), and oxygenation, and shorten both recovery time and hospitalization duration in patients with AECOPD. Nebulized budesonide, an inhaled corticosteroid (ICS), alone may be a suitable alternative to treat AECOPD in some patients, providing similar benefits to intravenous methylprednisolone. Using a bioinformatics method to analyze 16S data from AECOPD patients, Ma et al. showed that there were similar effects of ICS and SCS on the sputum microbiome in patients with AECOPD, with comparable effects on bacterial abundance and microecological diversity. Additionally, ICS has little effect on the lung microbiome of AECOPD, potentially a treatment advantage by reducing the risk of selecting for antibiotic resistant organisms.

Since 2018, there has been debate whether a new definition of AECOPD is needed. Some experts have proposed that the existing definition, based mainly on the patient's perception of symptoms, lacks specificity and objectivity. The articles in this special Research Topic contribute to the ongoing work to define predictive biomarkers for, and new etiological entities of, AECOPD. With continued worldwide attention, there is hope that breaking the current pattern of AECOPD is just down the road.

## Author Contributions

YC, JZ, and JC are responsible for the proposal of the Research Topic, calling the input for the special toolkits, editing the submitted manuscripts, and writing and revising the editorial. All authors contributed to the article and approved the submitted version.

## Funding

This work was supported by National Natural Science Foundation of China (Nos. 82090014 and 81970037) and Capital Health Development Research Project (2020-2Z-40917).

## Conflict of Interest

The authors declare that the research was conducted in the absence of any commercial or financial relationships that could be construed as a potential conflict of interest.

## Publisher's Note

All claims expressed in this article are solely those of the authors and do not necessarily represent those of their affiliated organizations, or those of the publisher, the editors and the reviewers. Any product that may be evaluated in this article, or claim that may be made by its manufacturer, is not guaranteed or endorsed by the publisher.
